# Nonadherence to Primary Prophylaxis against *Pneumocystis jirovecii* Pneumonia

**DOI:** 10.1371/journal.pone.0005002

**Published:** 2009-03-25

**Authors:** James D. Heffelfinger, Andrew C. Voetsch, Glenn V. Nakamura, Patrick S. Sullivan, A. D. McNaghten, Laurence Huang

**Affiliations:** 1 Division of HIV/AIDS Prevention, National Center for HIV/AIDS, Viral Hepatitis, STD and TB Prevention, Centers for Disease Control and Prevention, Atlanta, Georgia, United States of America; 2 Rollins School of Public Health, Emory University, Atlanta, Georgia, United States of America; 3 HIV/AIDS Division and Division of Pulmonary and Critical Care Medicine, University of California San Francisco, San Francisco, California, United States of America; University of Stellenbosch, South Africa

## Abstract

**Background:**

Despite the effectiveness of prophylaxis, *Pneumocystis jirovecii* pneumonia (PCP) continues to be the most common serious opportunistic infection among HIV-infected persons. We describe factors associated with nonadherence to primary PCP prophylaxis.

**Methodology/Principal Findings:**

We used 2000–2004 data from the Supplement to HIV/AIDS Surveillance (SHAS) project, a cross-sectional interview project of HIV-infected persons ≥18 years conducted in 18 states. We limited the analysis to persons who denied having prior PCP, reported having a current prescription to prevent PCP, and answered the question “In the past 30 days, how often were you able to take the PCP medication(s) exactly the way your doctor told you to take them?” We used multivariable logistic regression to describe factors associated with nonadherence. Of 1,666 subjects prescribed PCP prophylaxis, 305 (18.3%) were nonadherent. Persons were more likely to be nonadherent if they reported using marijuana (adjusted odds ratio [aOR] = 1.6, 95% confidence interval [CI] = 1.1–2.4), non-injection drugs other than marijuana (aOR = 1.5, 95% CI = 1.0–2.1), or injection drugs (aOR = 2.3, 95% CI = 1.3–4.1) in the past year; their mental health was “not good” for ≥1 day during the past month (aOR = 1.6, 95% CI = 1.2–2.2); their most recent CD4 count was <200 cells/μL (aOR = 1.6, 95% CI = 1.1–2.2); or taking ART usually (aOR = 9.6, 95% CI = 6.7–13.7) or sometimes/rarely/never (aOR = 18.4, 95% CI = 11.1–30.4), compared with always, as prescribed.

**Conclusion/Significance:**

Providers should inquire about and promote strategies to improve adherence to PCP prophylaxis, particularly among persons who use illicit drugs, have mental health issues, and who are not compliant with ART to reduce the occurrence of PCP.

## Introduction

*Pneumocystis jirovecii* pneumonia (PCP) continues to be the most common serious opportunistic infection occurring among persons with HIV infection in the United States despite effective prophylactic therapy [1]. During the 1980s, PCP was the AIDS-defining illness for approximately two-thirds of patients, and it was estimated that 75% of HIV-infected persons would develop PCP in their lifetime [2], [3]. Trimethoprim-sulfamethoxazole (TMP-SMX) was shown to be effective in preventing PCP in persons with AIDS in the late 1980s [4], and the proportion of cases in which PCP was an AIDS-defining illness decreased appreciably as prescription of TMP-SMX to prevent PCP became the standard of care [5], [6]. The widespread use of antiretroviral therapy (ART) in the late 1990s led to a further decline in the incidence of PCP [3]. Current Centers for Disease Control and Prevention (CDC) guidelines recommend the initiation of primary PCP prophylaxis for persons with CD4 cell counts less than 200 cells/μL or a history of oropharyngeal candidiasis, and discontinuation of prophylaxis for persons who have responded to ART with an increase in CD4 cell counts to more than 200 cells/μL for greater than 3 months [7].

Although prophylaxis against PCP is effective, the benefits can only be realized if HIV-positive persons access healthcare, providers appropriately prescribe prophylaxis to persons at risk for PCP, and patients adhere to prescribed PCP prophylaxis. In an analysis of PCP cases diagnosed during 1999–2001, 44% of cases occurred among persons not receiving care, 10% occurred among persons in care who met criteria for prophylaxis but were not prescribed prophylaxis, and 41% occurred among persons prescribed prophylaxis but who were either not adherent to treatment or who developed PCP despite appropriate use [3]. The U.S. Healthy People 2010 goal is for 95% of eligible persons to receive PCP prophylaxis [8]. The proportion of eligible persons prescribed PCP prophylaxis in a large U.S. cohort of HIV-positive persons was 80% [9], whereas in another study, the proportion of providers who adhered with guidelines for prescribing PCP prophylaxis in federally funded HIV treatment facilities ranged from 80–94% [10]. Lundberg and colleagues found that provider nonadherence to prophylaxis guidelines was uncommon and that patient nonadherence to prophylaxis was the most common reason for the occurrence of PCP [11]. Few studies have examined patient adherence to PCP prophylaxis and reasons for nonadherence. In one study, only 49% of persons reported taking at least 80% of their prescribed PCP prophylaxis in the previous seven days [12]. Presence of family, better mental health, and greater self-efficacy were associated with increased adherence to PCP prophylaxis, whereas injection drug use was associated with nonadherence [12].

The objective of this analysis was to identify factors associated with and self-reported reasons for nonadherence to primary PCP prophylaxis among HIV-infected persons.

## Methods

### Project

The Supplement to HIV and AIDS Surveillance (SHAS) project was a cross-sectional behavioral surveillance project of persons with HIV infection. The methods have been previously described [13]. In brief, adults (≥18 years) reported with HIV or AIDS through routine case surveillance were eligible for participation. Persons with HIV/AIDS were enrolled using one of two methods: 1) facility-based recruitment of all eligible persons seeking treatment at selected healthcare facilities in 13 cities in 9 states (Denver, Colorado; Hartford and New Haven, Connecticut; Jacksonville, Miami, and Tampa, Florida; Atlanta, Georgia; Chicago, Illinois; Baltimore, Maryland; Detroit, Michigan; Jersey City and Paterson, New Jersey; and Philadelphia, Pennsylvania); and 2) population-based recruitment of all eligible persons in 5 states and in 6 cities and one county in four additional states (Phoenix and Tucson, Arizona; Delaware; Kansas; Los Angeles County, California; Minneapolis/St. Paul, Minnesota; New Mexico; South Carolina; Austin and Houston Texas; and Washington). Most SHAS participants were enrolled in the project soon after they began to receive care for HIV infection. Informed consent was obtained by having the participant read and sign the informed consent form, having the interviewer read the form to the participant and asking the participant to sign the form, or having the interviewer read the form to the participant and indicating on the form that participant provided verbal consent. The project received institutional review board approval at both the CDC and local levels.

### Measures

From May 2000 through June 2004, SHAS participants were asked about medication history, adherence to medications, and reasons for nonadherence. SHAS has no way to measure adherence with medications independently, but instead relies on self-reported adherence. To determine if respondents were taking PCP prophylaxis and the specific medication(s), they were asked to look at a list of PCP medications on a card. After indicating those medications that were ever prescribed to them, they were asked to indicate which ones they were currently taking. Because there were no questions that specifically addressed whether respondents were taking PCP in this analysis because survey questions did not allow us to determine whether a current prescription for PCP medication was for treatment or secondary prophylaxis against PCP. We limited our analysis to persons who answered the question “Sometimes it is difficult to take medications for many reasons. In the past 30 days, how often were you able to take your PCP medication(s) exactly the way your doctor told you to take them?” Persons answering “always” were considered adherent and those answering “usually”, “sometimes”, or “rarely or never” were considered nonadherent to PCP prophylaxis; persons answering “unknown” to this question and those who did not answer this question were excluded from the analysis because we did not have complete information about their adherence to PCP prophylaxis. Nonadherent persons were asked about the primary reason for nonadherence by asking, “What are some of the reasons why you don't take your PCP medicine(s) as the doctor prescribed?” Respondents were able to provide as many as three reasons for nonadherence. We coded the responses into three categories: 1) side effects; 2) difficulties with access to care or obtaining/taking medications; and 3) problems with scheduling, memory, or lack of perceived necessity. Region of residence was defined by the U.S. Census Bureau. Risk for recent alcohol abuse was defined as answering “yes” to two or more CAGE questions [14] and reporting alcohol use in the previous year. The daily pill burden was calculated by adding the number of medications prescribed for ART, prophylaxis against opportunistic infections, and other conditions. Assessment of mental health during the previous month was determined by the answer to the question, “Now thinking about your mental health, which includes stress, depression, and problems with emotions, for how many days during the past 30 days was your mental health not good?” Respondents were asked to look at a list of “HIV/AIDS medicines” (ART medications) and tell the interviewer which of the listed medications they had ever taken and which they were currently taking. Adherence to ART was defined for persons who reported having a current prescription for ART and who answered the question “In the past 30 days, how often were you able to take your HIV/AIDS medicines exactly the way your doctor told you to take them?” Persons could answer “always”, “usually”, “sometimes”, “rarely or never”, or “unknown”, and their adherence to ART medicines was categorized accordingly, although those answering “sometimes” or “rarely or never” were grouped together for this analysis. Illicit drug use was categorized as no drug use, use of marijuana only, use of non-injection drugs other than marijuana, and use of injection drugs in the past year. In addition, for some analyses, injection drug use was categorized as injection drug use versus no injection drug use in the past year.

### Analyses

Statistical testing for differences (p<0.05) between factors associated with adherence and nonadherence to PCP prophylaxis was performed using chi-squared or Fisher's exact test, as appropriate. We used multivariable logistic regression models using STATA version 8.0 (STATA Corporation, Corpus Christi, TX) to calculate adjusted odds ratios and 95% confidence intervals for factors associated with nonadherence to PCP prophylaxis. Because region of residence and year of SHAS interview were identified as likely confounding factors of nonadherence to PCP prophylaxis a priori, these variables were included in all multivariable models. In multivariable analyses, controlling for region of residence and year of SHAS interview, preliminary models included factors that were associated (*p*≤0.10) with nonadherence to primary PCP prophylaxis in bivariate analyses. We performed stepwise elimination to remove factors that were not significantly associated (*p*>0.05) with nonadherence [15]. We assessed all possible two-way interactions between variables using a forward selection model using SAS version 9.1 (SAS Institute, Cary, NC), with a p-value for entry that was corrected for multiple comparisons between the number of possible interaction terms. We conducted a sensitivity analysis using a more restrictive definition of nonadherence (having only sometimes, rarely, or never taken PCP medications exactly as prescribed during the past month). Because the most important indication for PCP prophylaxis is a low CD4 cell count, we also conducted multivariable analysis after restricting the data set to persons with CD4 counts less than 200 cells/μL.

## Results

**Figure 1:**
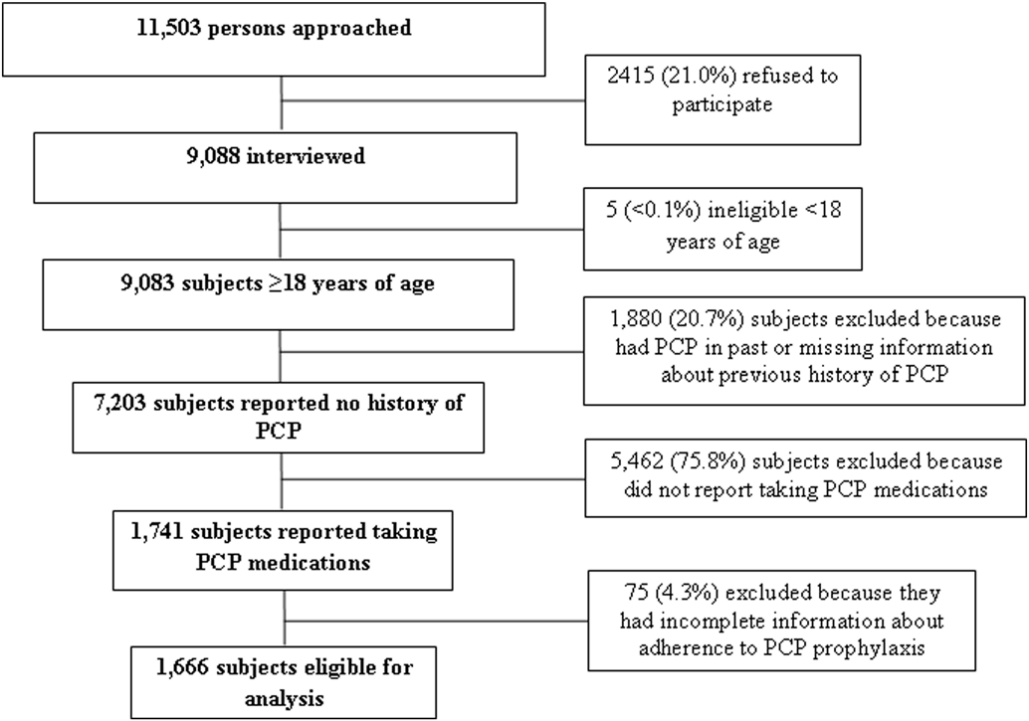
Eligibility and selection criteria for subjects included in this analysis of nonadherence to *Pneumocystis jirovecii* pneumonia (PCP) prophylaxis – Supplement to HIV/AIDS Surveillance (SHAS) project, 2000–2004.

Of the 11,503 persons asked to participate in SHAS, 1,666 (18.3% of interviewed) respondents who had a prescription for primary PCP prophylaxis and complete information about adherence to PCP prophylaxis were included in the analysis. Eligibility and selection criteria for subjects included in this analysis of nonadherence to PCP prophylaxis are summarized in Figure 1. Among these 1,666 persons, 76.8% were male, 75.4% considered themselves to be non-Hispanic black or Hispanic, and the median age was 40 (range: 19–75) years. Seventy-nine percent of subjects who reported having a current prescription for PCP medications were prescribed TMP-SMX.

Three hundred five (18.3%) respondents were nonadherent with primary PCP prophylaxis during the month preceding their interview. On bivariate analysis, nonadherence to primary PCP prophylaxis was associated with year of SHAS interview, illicit drug use in the past year, current risk for alcohol abuse, description of mental health as “not good” for ≥1 day in the past month, most recent CD4 count <200 cells/μL, most recent HIV viral load >5,000 copies/mL, and nonadherence to ART in the past month (Table 1). Sex, age group, race/ethnicity, income, having health insurance, education, U.S. region of residence, living situation, current PCP medication(s) prescribed, daily pill burden, and type of recruitment were not associated with nonadherence to PCP prophylaxis.

**Table 1:** Characteristics of 1,666 persons with HIV infection prescribed primary prophylaxis against *Pneumocystis jirovecii* pneumonia (PCP), by nonadherence to prophylaxis – Supplement to HIV/AIDS Surveillance (SHAS) project, 2000–2004.

Characteristics	Primary PCP Prophylaxis	
	Nonadherent*	Adherent*
	N = 305	N = 1,361
	n (%)	n (%)
Year of SHAS interview†		
2000	45 (14.7)	111 (8.2)
2001	82 (26.9)	407 (29.9)
2002	93 (30.5)	411 (30.2)
2003	60 (19.7)	333 (24.5)
2004	25 (8.2)	99 (7.3)
Sex		
Male	240 (78.7)	1040 (76.4)
Female	65 (21.3)	321 (23.6)
Race/Ethnicity		
White, non-Hispanic	67 (22.0)	273 (20.1)
Black, non-Hispanic	179 (58.7)	762 (56.6)
Hispanic	46 (15.1)	269 (19.8)
Other	13 (4.3)	57 (4.7)
Age group (years)		
18–29	38 (12.5)	126 (9.3)
30–39	112 (40.0)	498 (36.6)
40–49	113 (37.0)	550 (40.4)
≥50	32 (10.5)	187 (13.7)
Employed		
Yes	92 (30.2)	383 (28.1)
No	213 (69.8)	978 (71.9)
Current annual income		
<$10,000	161 (57.8)	666 (48.9)
$10,000–20,000	53 (17.4)	265 (19.5)
≥$20,000	70 (22.9)	322 (23.7)
Unknown or refused to answer	21 (6.9)	108 (7.9)
Education		
<High school/General Equivalency Diploma (GED)	94 (30.8)	446 (32.8)
High school/GED	95 (31.1)	433 (31.8)
>High school/GED	116 (38.0)	482 (35.4)
Medical insurance		
Yes	240 (78.7)	1,075 (79.0)
No	65 (21.3)	286 (21.0)
Region of residence‡		
Northeast	25 (8.2)	73 (5.4)
South	155 (50.8)	728 (53.5)
Midwest	48 (15.7)	212 (15.6)
West	77 (25.2)	348 (25.6)
Current living situation		
Alone	100 (32.8)	403 (29.6)
With partner, family, or friend	172 (56.4)	837 (61.5)
In a medical facility	10 (3.3)	42 (3.1)
In shelter	15 (4.9)	47 (3.4)
Other±	8 (2.6)	32 (2.3)
Illicit drug use in past year§ †		
No	142 (46.6)	899 (66.0)
Marijuana only	54 (17.7)	179 (13.1)
Non-injection drugs other than marijuana	81 (26.6)	233 (17.1)
Injection drug use	28 (9.2)	50 (3.7)
Risk for alcoholism|| †		
Yes	103 (33.8)	328 (24.1)
No	202 (66.2)	1.033 (75.9)
Mental health described as not good ≥1 day during the last month†		
Yes	232 (76.1)	821 (60.3)
No	73 (23.9)	540 (39.7)
Recent CD4 count (cells/μL)†		
<200	160 (52.5)	536 (39.4)
≥200	77 (25.2)	393 (28.9)
Unknown/missing	68 (22.3)	432 (31.7)
Viral load (copies/mL)†		
≤5000	108 (35.4)	525 (38.6)
>5,000	74 (24.3)	240 (17.6)
Unknown/missing	123 (40.3)	596 (43.8)
Current PCP medication prescribed		
Trimethoprim-sulfamethoxazole	243 (79.7)	1078 (79.2)
Dapsone	37 (12.1)	169 (12.4)
Other/unknown	25 (8.2)	114 (8.4)
Antiretroviral therapy (ART) in the past month†		
On ART, always adherent	59 (19.3)	910 (66.9)
On ART, usually adherent	125 (41.0)	194 (14.2)
On ART, sometimes, rarely, or never adherent	59 (19.3)	44 (3.2)
On ART, unknown or missing information about adherence	20 (6.6)	104 (7.6)
Not on ART	42 (13.8)	109 (8.0)
Total number of pills prescribed for daily use		
1–3	87 (28.5)	371 (27.3)
4–5	120 (39.3)	515 (37.8)
≥6	98 (32.1)	475 (34.9)
Recruitment type		
Facility-based	192 (62.9)	894 (65.7)
Population-based	113 (37.0)	467 (34.3)

* Column percentages may not total 100% because of rounding.

† P-value <0.05 when comparing persons nonadherent with those adherent to PCP prophylaxis.

‡ Regions defined by U.S. Census Bureau; for SHAS sites included in this analysis, Northeast comprises NJ, CT, and PA; South comprises GA, MD, FL, SC, DE, and TX; Midwest comprises IL, KS, MI, and MN; and West comprises AZ, CA, CO, NM, and WA.

± Other responses included correctional institution, refused to answer, and other responses which did not fit into the listed response categories.

§ Categories are mutually exclusive.

|| Defined as answering yes to at least 2 of the CAGE screening questions and reporting alcohol use in the past year.

Multivariable analysis was performed to identify factors that were independently associated with nonadherence to primary PCP prophylaxis. Controlling for region of residence and year of interview, factors that were independently associated with nonadherence to primary PCP prophylaxis were use of marijuana, non-injection drugs other than marijuana, and injection drugs in the past year; description of mental health as not good for ≥1 day in the past month; most recent CD4 count <200 cells/μL; and having taken ART usually or sometimes/rarely/never as prescribed, having taken ART but having unknown or missing information about adherence to ART, or not having taken ART in the past month (Table 2). No significant interaction was found. The risk of nonadherence to PCP prophylaxis increased with the level of nonadherence to ART, being approximately twice as high among persons who reported having sometimes/rarely/never been adherent with ART as it was among those who reported having usually been adherent with ART during the preceding month.

**Table 2:** Multivariable analysis of factors associated with nonadherence to primary prophylaxis against *Pneumocystis jirovecii* pneumonia (PCP) – Supplement to HIV/AIDS Surveillance (SHAS) project, 2000–2004.

Characteristics	Adjusted Odds Ratio (95% Confidence Interval)*
	N = 1,666
Illicit drug use in last year	
No	Reference
Marijuana only	1.6 (1.1–2.4)
Non-injection drugs other than marijuana	1.5 (1.0–2.1)†
Injection drug use	2.3 (1.3–4.1)
Number of days mental health described as not good last month	
0	Reference
≥1	1.6 (1.2–2.2)
Recent CD4 count (cells/μL)	
<200	1.6 (1.1–2.2)
≥200	Reference
Unknown/missing	0.9 (0.6–1.4)
Antiretroviral therapy (ART) in the past month	
On ART, always adherent	Reference
On ART, usually adherent	9.6 (6.7–13.7)
On ART, sometimes, rarely, or never adherent	18.4 (11.1–30.4)
On ART, unknown or missing information about adherence	2.7 (1.5–4.7)
Not taking ART	5.1 (3.3–8.1)

* In addition to adjusting for variables included in this table, we controlled for region of residence in the U.S. and year of SHAS interview.

† P-value<0.05.

Limiting the multivariable analysis to persons whose most recent CD4 count was <200 cells/μL and controlling for region of residence and year of interview, injection drug use in the past year (adjusted odds ratio [aOR]: 2.3; 95% confidence interval [CI]: 1.1–4.9) and having taken ART usually (aOR: 9.2; 95% CI: 5.5–15.5) or sometimes/rarely/never (aOR: 26.8; 95% CI: 13.1–54.5) as prescribed, having taken ART but having unknown or missing information about adherence to ART (aOR: 2.6; 95% CI: 1.2–5.3), or not having taken ART (aOR: 4.8; 95% CI: 2.6–9.1) in the past month were independently associated with nonadherence to primary PCP prophylaxis. The risk of nonadherence to PCP prophylaxis was almost three times as high among persons who reported having sometimes/rarely/never been adherent with ART as it was among those who reported having usually been adherent with ART during the preceding month.

When we used the more restrictive definition of nonadherence to PCP prophylaxis during the past month (i.e., defining it as taking PCP medications sometimes, rarely, or never), our findings on multivariable analysis did not change appreciably (data not shown). However, description of mental health as not good for ≥1 day in the past month did not remain in the model and injection drug use during the past year was no longer an independent risk factor for nonadherence using the more restrictive definition of nonadherence.

Of the 305 persons who reported that they did not take primary PCP prophylaxis exactly as prescribed during the month before interview, 264 (86.6%) reported the primary reason why they did not do so. Side effects were cited by 21.6%; difficulties with access to care or obtaining/taking medications were reported by 13.6%; and problems with scheduling, difficulty remembering to take the medication(s), or lack of perceived necessity were listed by 64.8% of subjects (Table 3). The most common reason cited for not taking PCP prophylaxis was forgetting to take it.

**Table 3:** Primary reason for not taking medications to prevent *Pneumocystis jirovecii* pneumonia (PCP) listed by 264 persons who were nonadherent with primary PCP prophylaxis – Supplement to HIV/AIDS Surveillance (SHAS) project, 2000–2004.

Primary Reason for Not Taking PCP Medications as Prescribed	Nonadherent with Primary PCP Prophylaxis
	N = 264
	n (%)
**Side effects**	**57 (21.6)**
**Limited access, mental health, or living situation**	**36 (13.6)**
Cannot afford medications	2 (0.8)
Do not understand either how to take medications or why they were prescribed	3 (1.1)
Cannot get to clinic or physician's office	21 (8.0)
“Back on street”	4 (1.5)
Depressed	4 (1.5)
In jail or prison	2 (0.8)
**Issues with scheduling, memory, or lack of perceived necessity**	**171 (64.8)**
Cannot fit taking medications into schedule	35 (13.3)
Often forget to take medications	119 (45.1)
“Take too many medications”	6 (2.3)
Do not believe in them or do not believe they need to take them	11 (4.2)

## Discussion

In a large cohort of persons living with HIV infection, we found that almost one-fifth were nonadherent to primary PCP prophylaxis. We found that nonadherence to PCP prophylaxis was associated with illicit drug use, mental health issues including depression, nonadherence to prescribed ART, and low CD4 cell count.

Our findings regarding illicit drug use are consistent with those of several studies evaluating adherence to ART [16], [17], [18], [19], [20], [21], [22], [23], however current drug use was not found to be an independent risk factor in either study evaluating risk factors for nonadherence to PCP prophylaxis [11], [12]. We did not find nonadherence to be associated with age, race/ethnicity, or daily pill burden, factors which have previously been reported to be associated with increased risk for nonadherence to PCP prophylaxis and/or ART.

Consistent with our findings, Eldred and colleagues found that a high Mental Health Inventory-5 score, which indicates better mental health, was associated with adherence to PCP prophylaxis [12]. Lundberg and colleagues found that persons with current psychiatric illness were more likely to be nonadherent to PCP prophylaxis; however they did not find this condition to be an independent risk factor for nonadherence [11]. Additionally, mental health issues have been associated with nonadherence to ART in several studies [16], [23], [24], [25], [26]. It is possible, though, that persons with better mental health may report adherence to medications more accurately than persons with poorer mental health.

Persons who reported being nonadherent to primary PCP prophylaxis were much more likely to report also being nonadherent to ART, which is consistent with the findings of Lundberg and associates [11]. We found that the risk of nonadherence to PCP prophylaxis was highest among those with the highest levels of reported nonadherence to ART medications.

We found that low CD4 cell count was independently associated with nonadherence to PCP prophylaxis. However, 29% of respondents reported having recent CD4 cell counts that were ≥200 cells/μL, and 32% could not provide information about their most recent CD4 cell count. Respondents whose most recent CD4 cell counts were ≥200 cells/μL may have been started on PCP prophylaxis based on a declining trend in CD4 cell counts, may not have had CD4 cell counts that were ≥200 cells/μL for at least 3 months, or may not have accurately recalled their most recent CD4 count. When we limited our analysis to persons who reported that their most recent CD4 cell count was <200 cells/μL, our findings did not change appreciably. Among subjects reporting they had a CD4 count <200 cells/μL, recent injection drug use and nonadherence to ART remained independent predictors for nonadherence to PCP prophylaxis; however, non-injection drug use did not remain an independent risk factor.

Although several studies have found that nonadherence to PCP prophylaxis and/or ART was associated with age [19], [23], [24], [25] or race/ethnicity [11], [23], we did not find nonadherence to PCP prophylaxis to be independently associated with these or other sociodemographic characteristics. In addition, we found that daily pill burden was not associated with nonadherence to PCP prophylaxis, which contradicts the findings of several studies that have found that an increased number of ART pills taken daily is associated with nonadherence to PCP prophylaxis or ART [11], [23], [24]. Our findings may differ from those of other investigators because we were able to control more completely for potentially confounding variables or, with respect to the number of pills taken daily, because we used a different measure for pill burden that included all medications prescribed and not just ART medications.

Forgetting to take PCP prophylaxis was the most common reason provided by participants in our analysis, followed by concern about side effects and inability to fit taking the medication(s) into one's schedule, which parallels reasons given for nonadherence to ART among SHAS participants in a separate analysis [23]. Forgetting to take medications has previously been reported to be an important reason for nonadherence to PCP prophylaxis [11], ART [27], and prescribed therapy for other conditions [28], [29]. Structural barriers such as limited access to healthcare or medications were not prominent as reasons for nonadherence in our analysis.

This study has several imitations. Because this was an interview study, our findings are subject to recall and social desirability bias [30]. However, asking subjects about their adherence to primary PCP prophylaxis in the month before the interview should have reduced recall bias. Because social desirability would be expected to lead subjects to underreport nonadherence to medications, our estimate may be a minimum one [31]. Because hospitals that participated in areas which used facility-based recruitment may not have been representative of all facilities that provide care to HIV-infected persons in these areas, and persons recruited and interviewed by SHAS sites were not representative of all HIV-infected persons, or of all HIV-infected persons in care, our findings are subject to selection bias. In addition, limited or no information was collected in the interview about a number of factors that may be important for nonadherence, including social support, isolation, treatment efficacy, and self-efficacy [32], [33]. Despite these limitations, the SHAS surveillance system represents the largest survey of HIV-infected persons in the U.S. and therefore has the most complete data to assess self-reported adherence to PCP prophylaxis.

Nonadherence to PCP prophylaxis is an important contributor to adverse clinical events, and nonadherence to relatively simple regimens used to protect against PCP suggests that there may be greater challenges with adherence to ART regimens, which are more complicated [11]. We found that an appreciable proportion of HIV-infected persons were nonadherent to prescribed medications to prevent PCP, and that persons who were nonadherent to PCP prophylaxis were much more likely to be nonadherent to ART. Providers should inquire about adherence to prescribed medications to prevent PCP at every HIV care visit, paying particular attention to persons who use illicit drugs, have mental health issues, have low CD4 cell counts, and are nonadherent to ART or medications prescribed for other conditions. These variables should be triggers for providers to consider the complex issue of adherence. Providers should take every opportunity to educate their patients about the importance of compliance with ART and medications to prevent opportunistic infections, discuss the possible side effects of prescribed medications, and assist patients about ways to remember to take their medications as prescribed. In addition, providers should regularly ask HIV-infected patients about illicit drug use and mental health issues and treat or refer persons with substance abuse and/or mental illnesses to ensure optimal adherence to prescribed medications. Although few studies have evaluated factors associated with nonadherence or ways to improve adherence to medications to prevent PCP, the commonality of associated factors for nonadherence to PCP prophylaxis and ART suggests that interventions for ART adherence may be also be used to improve adherence to PCP prophylaxis. Studies evaluating adherence to ART, prophylaxis against PCP and other opportunistic infections, and prescribed medications for other conditions may shed light on how to improve adherence and quality of life and reduce morbidity and mortality among HIV-infected persons.

## References

[1] Kaplan JE, Hanson D, Dworkin MS, Frederick T, Bertolli J (2000). Epidemiology of human immunodeficiency virus-associated opportunistic infections in the United States in the era of highly active antiretroviral therapy.. Clin Infect Dis.

[2] Hay JW, Osmond DH, Jacobson MA (1988). Projecting the medical costs of AIDS and ARC in the United States.. J Acquir Immune Defic Syndr.

[3] Morris A, Lundgren JD, Masur H, Walzer PD, Hanson DL (2004). Current epidemiology of Pneumocystis pneumonia.. Emerg Infect Dis.

[4] Fischl MA, Dickinson GM, La Voie L (1988). Safety and efficacy of sulfamethoxazole and trimethoprim chemoprophylaxis for Pneumocystis carinii pneumonia in AIDS.. JAMA.

[5] CDC (1990). HIV/AIDS Surveillance Report, 1989.

[6] CDC (1993). HIV/AIDS Surveillance Report, 1992.

[7] Kaplan JE, Masur H, Holmes KK (2002). Guidelines for preventing opportunistic infections among HIV-infected persons–2002. Recommendations of the U.S. Public Health Service and the Infectious Diseases Society of America.. MMWR Recomm Rep.

[8] US Department of Health and Human Services (2000). Healthy People 2010: understanding and improving health. 2nd ed. 2000.

[9] Teshale EH, Hanson DL, Wolfe MI, Brooks JT, Kaplan JE (2007). Reasons for lack of appropriate receipt of primary Pneumocystis jiroveci pneumonia prophylaxis among HIV-infected persons receiving treatment in the United States: 1994–2003.. Clin Infect Dis.

[10] Kaplan JE, Parham DL, Soto-Torres L, van Dyck K, Greaves JA (1999). Adherence to guidelines for antiretroviral therapy and for preventing opportunistic infections in HIV-infected adults and adolescents in Ryan White-funded facilities in the United States.. J Acquir Immune Defic Syndr.

[11] Lundberg BE, Davidson AJ, Burman WJ (2000). Epidemiology of Pneumocystis carinii pneumonia in an era of effective prophylaxis: the relative contribution of non-adherence and drug failure.. AIDS.

[12] Eldred LJ, Wu AW, Chaisson RE, Moore RD (1998). Adherence to antiretroviral and pneumocystis prophylaxis in HIV disease.. J Acquir Immune Defic Syndr Hum Retrovirol.

[13] Buehler JW, Diaz T, Hersh BS, Chu SY (1996). The supplement to HIV-AIDS Surveillance project: an approach for monitoring HIV risk behaviors.. Public Health Rep.

[14] Maisto SA, Saitz R (2003). Alcohol use disorders: screening and diagnosis.. Am J Addict.

[15] Agresti A (2002). Categorical Data Analysis. (Second edition)..

[16] Ammassari A, Antinori A, Aloisi MS, Trotta MP, Murri R (2004). Depressive symptoms, neurocognitive impairment, and adherence to highly active antiretroviral therapy among HIV-infected persons.. Psychosomatics.

[17] Arnsten JH, Demas PA, Grant RW, Gourevitch MN, Farzadegan H (2002). Impact of active drug use on antiretroviral therapy adherence and viral suppression in HIV-infected drug users.. J Gen Intern Med.

[18] Berg KM, Demas PA, Howard AA, Schoenbaum EE, Gourevitch MN (2004). Gender differences in factors associated with adherence to antiretroviral therapy.. J Gen Intern Med.

[19] Howard AA, Arnsten JH, Lo Y, Vlahov D, Rich JD (2002). A prospective study of adherence and viral load in a large multi-center cohort of HIV-infected women.. AIDS.

[20] Lafeuillade A (2001). Factors affecting adherence and convenience in antiretroviral therapy.. Int J STD AIDS.

[21] Martini M, Recchia E, Nasta P, Castanotto D, Chiaffarino F (2004). Illicit drug use: can it predict adherence to antiretroviral therapy?. Eur J Epidemiol.

[22] Moss AR, Hahn JA, Perry S, Charlebois ED, Guzman D (2004). Adherence to highly active antiretroviral therapy in the homeless population in San Francisco: a prospective study.. Clin Infect Dis.

[23] Sullivan PS, Campsmith ML, Nakamura GV, Begley EB, Schulden J (2007). Patient and regimen characteristics associated with self-reported nonadherence to antiretroviral therapy.. PLoS ONE.

[24] Carrieri MP, Leport C, Protopopescu C, Cassuto JP, Bouvet E (2006). Factors associated with nonadherence to highly active antiretroviral therapy: a 5-year follow-up analysis with correction for the bias induced by missing data in the treatment maintenance phase.. J Acquir Immune Defic Syndr.

[25] Gordillo V, del Amo J, Soriano V, Gonzalez-Lahoz J (1999). Sociodemographic and psychological variables influencing adherence to antiretroviral therapy.. AIDS.

[26] Starace F, Ammassari A, Trotta MP, Murri R, De Longis P (2002). Depression is a risk factor for suboptimal adherence to highly active antiretroviral therapy.. J Acquir Immune Defic Syndr.

[27] Samet JH, Libman H, Steger KA, Dhawan RK, Chen J (1992). Compliance with zidovudine therapy in patients infected with human immunodeficiency virus, type 1: a cross-sectional study in a municipal hospital clinic.. Am J Med.

[28] Haynes RB, Taylor DW, Sackett DL, Gibson ES, Bernholz CD (1980). Can simple clinical measurements detect patient noncompliance?. Hypertension.

[29] Sumartojo E (1993). When tuberculosis treatment fails. A social behavioral account of patient adherence.. Am Rev Respir Dis.

[30] Sackett DL (1979). Bias in analytic research.. J Chronic Dis.

[31] Murri R, Ammassari A, Trotta MP, De Luca A, Melzi S (2004). Patient-reported and physician-estimated adherence to HAART: social and clinic center-related factors are associated with discordance.. J Gen Intern Med.

[32] Altice FL, Mostashari F, Friedland GH (2001). Trust and the acceptance of and adherence to antiretroviral therapy.. J Acquir Immune Defic Syndr.

[33] Holmes WC, Pace JL (2002). HIV-seropositive individuals' optimistic beliefs about prognosis and relation to medication and safe sex adherence.. J Gen Intern Med.

